# miR-182-5p and miR-378a-3p regulate ferroptosis in I/R-induced renal injury

**DOI:** 10.1038/s41419-020-03135-z

**Published:** 2020-10-28

**Authors:** Chenguang Ding, Xiaoming Ding, Jin Zheng, Bo Wang, Yang Li, Heli Xiang, Meng Dou, Yuxi Qiao, Puxun Tian, Wujun Xue

**Affiliations:** 1 Department of Kidney Transplantation, Nephropathy Hospital, The First Affiliated Hospital of Xi’an Jiaotong University, Xi’an, 710061 China; 2 Institute of Organ Transplantation, Xi’an Jiaotong University, Xi’an, 710061 China; 3 Department of Materials Science and Engineering, Monash University, Clayton, Australia

**Keywords:** Cancer therapy, DNA damage and repair

## Abstract

Renal tubular cell death is the key factor of the pathogenesis of ischemia/reperfusion (I/R) kidney injury. Ferroptosis is a type of regulated cell death (RCD) found in various diseases. However, the underlying molecular mechanisms related to ferroptosis in renal I/R injury remain unclear. In the present study, we investigated the regulatory role of microRNAs on ferroptosis in I/R-induced renal injury. We established the I/R-induced renal injury model in rats, and H/R induced HK-2 cells injury in vitro. CCK-8 was used to measure cell viability. Fe^2+^ and ROS levels were assayed to evaluate the activation of ferroptosis. We performed RNA sequencing to profile the miRNAs expression in H/R-induced injury and ferroptosis. Western blot analysis was used to detect the protein expression. qRT-PCR was used to detect the mRNA and miRNA levels in cells and tissues. We further used luciferase reporter assay to verify the direct targeting effect of miRNA. We found that ischemia/reperfusion-induced ferroptosis in rat’s kidney. We identified that miR-182-5p and miR-378a-3p were upregulated in the ferroptosis and H/R-induced injury, and correlates reversely with glutathione peroxidases 4 (GPX4) and solute carrier family 7 member 11 (SLC7A11) expression in renal I/R injury tissues, respectively. In vitro studies showed that miR-182-5p and miR-378a-3p induced ferroptosis in cells. We further found that miR-182-5p and miR-378a-3p regulated the expression of GPX4 and SLC7A11 negatively by directly binding to the 3′UTR of GPX4 and SLC7A11 mRNA. In vivo study showed that silencing miR-182-5p and miR-378a-3p alleviated the I/R-induced renal injury in rats. In conclusion, we demonstrated that I/R induced upregulation of miR-182-5p and miR-378a-3p, leading to activation of ferroptosis in renal injury through downregulation of GPX4 and SLC7A11.

## Introduction

Acute renal failure (ARF) is characterized by the sustained loss of kidney function, of which the primary cause is ischemia/reperfusion (I/R) kidney injury^1^. I/R injury is prone to occur after kidney transplantation, leading to the failure of the transplanted kidney^2–4^. The pathogenesis and mechanism of I/R injury is complicated. Now, it is well recognized that the death of renal tubular cells, resulting in the structural damage of renal tubules, is the main pathogenesis of I/R-induced renal injury^5,6^. The structural damage of renal tubules is the crucial and initial factors of the occurrence of ARF by reducing of glomerular filtration rate (GFR) and increasing interstitial edema^7^. However, the molecular mechanism of I/R-induced death of renal tubular cells is still not fully understood^8,9^.

Ferroptosis is a type of regulated cell death (RCD), which is different from other forms of cell death characterized by the accumulation of iron, lipid peroxidation, and condensed mitochondrial membrane densities^10^. Recently, ferroptosis has been found to participate in many disease processes, such as cancer development, myocardial infarction, and neurological disease^11–14^. Lots of genes regulate the process of ferroptosis by regulating the lipid peroxidization state and iron level of cells^15^. Glutathione peroxidases 4 (GPX4) inhibits ferroptosis by decreasing the lipid peroxidization in cells^16^. Solute carrier family 7 member 11 (SLC7A11) decreases the lipid peroxidization level of cells to inhibit ferroptosis by transporting the cystine into the cytosol to promote the production of GSH^17^. Acyl-CoA synthetase long-chain family member 4 (ACSL4) dictates ferroptosis sensitivity by shaping cellular lipid composition^18^. Recent studies have revealed that ferroptosis plays an important role in rhabdomyolysis-associated renal damage and severe acute pancreatitis (SAP) induced acute kidney injury^19,20^. Ferroptosis mediates postischemic and toxic renal necrosis, which may be therapeutically targeted by ferrostatins^21^, indicating that ferroptosis may play central roles in the pathogenesis of renal I/R injury.

MicroRNAs (miRNAs) are a class of small non-coding RNAs which downregulate gene expression post-transcriptionally by targeting the 3′UTR of mRNA^22^. Often, miRNAs function in numerous biological processes, including immune responses, autophagy, cell proliferation, differentiation, and apoptosis by means of miRNA-mRNA interaction^23–27^. Recently, more and more studies have found that miRNAs could also play their functional roles by cooperating with other non‐coding RNAs^12,28^. In T-ALL cells, hsa‐miR‐20b‐5p and hsa‐miR‐363‐3p modulated the survival of cells by affecting the expression of PTEN and BIM tumor suppressor genes^29^. In human gastric cancer cells, miR-15b and miR-16 modulate multidrug resistance by targeting BCL2^30^. Although microRNA could regulate the progression of I/R-induced renal injury by changing related gene expression^31^, whether multiple miRNAs could function synergistically in the ferroptosis in I/R kidney injury remains unknown. Here we identified that miR-182-5p and miR-378a-3p were upregulated in the I/R-induced renal injury to induce the occurrence of ferroptosis combinedly through downregulating the expression of GPX4 and SLC7A11.

## Materials and methods

### Cell culture, transfection, and H/R treatment

The human renal proximal tubular cell line HK-2 and mouse kidney epithelial cell line TCMK-1 were purchased from American Type Culture Collection (Manassas, VA, USA). The cells were cultured in Dulbecco’s modified Eagle’s medium (ThermoFisher, USA) supplemented with 10% fetal bovine serum, 100 U/mL penicillin, and 100 μg/mL streptomycin in a 37 °C incubator with humidified air containing 5% CO_2_. For the construction of I/R model in vitro, hypoxia/reoxygenation (H/R) of HK-2 cells and TCMK-1 cells was used to mimic the I/R in vitro as described in the previous study^32^. HK-2 cells were exposed to hypoxia (1% O_2_, 5% CO_2_, and 94% N_2_) for 24 h followed by 12 h of reoxygenation (21% O_2_, 5% CO_2_, and 74% N_2_). For the cell transfection, cells were transfected with either miR-182-5p and miR-378a-3p mimic or inhibitor (Ribobio, Guangzhou, China) by using Lipofectamine 3000 reagents (ThermoFisher, USA) or treated with GPX4 lentivirus or SLC7A11 lentivirus (Genepharma, Shanghai, China) for 24 h before the H/R treatment. The sequences of the mimic or inhibitors used were as follows: miR-182-5p mimic (sequence: 5′-UUUGGCAAUGGUAGAACUCACACU-3′), inhibitor (sequence: 5′-AGUGUGAGUUCUACCAUUGCCAAA-3′); miR-378a-3p mimic (sequence: 5′-ACUGGACUUGGAGUCAGAAGG-3′), inhibitor (sequence: 3′-CCUUCUGACUCCAAGUCCAGU-3′).

### Cell viability assay

The HK-2 cell viability assay was plated in a 96-well plate at a density of 5000 cells per well. For the detection of the role of ferroptosis in H/R-induced cells injury, the cells were treated with 1 μM ferrostatin-1 (Fer-1), 10 μM erastin or not 1 h before the H/R induction. For the detection of the role of miRNA in H/R-induced cells injury, the miR-182-5p mimics and miR-378a-3p mimics or inhibitor were transfected to cells 12 h before the H/R induction. The cells were detected the cell viability by using the Cell Counting Kit-8 cell viability kit according to the manufacturer’s instructions. The absorbance at 450 nm was measured using a microplate reader (SpectraMax i3x, Molecular Devices).

### Measurement of Fe^2+^, mitosox intensity, and ROS

For the iron detection, the kidney tissues or the cells collected were immediately homogenized with phosphate-buffered saline (PBS). After centrifugation, the supernatant was performed iron concentration (Fe^2+^ level) detection by using the Iron Assay Kit (Abcam, Shanghai, China) according to the manufacturer’s instruction. For ROS detection, cells were incubated with 10 μM DCFH-DA probe (Beyotime, Shanghai, China) for 25 min. Cells were then rinsed twice with PBS, and fluorescence was assayed with a fluorescence microplate reader at the excitation/emission 488/525 nm. Mitosox intensity was used to evaluate the mitochondrial superoxide by using the fluorescent MitoSox probe (Thermofisher, Shanghai, China). Cells were incubated in Hank’s buffer with 5 μM MitoSox-Red for 30 min in a 37 °C incubator with humidified air containing 5% CO_2_. Then the cells were washed with PBS and the mitosox intensity were assessed by using the microplate reader at the absorbance length of 488 nm (SpectraMax i3x, Molecular Devices). Thresholds were adjusted by using nonstained and stained cells for MitoSox-Red fluorescence.

### Measurement of cell death

Annexin V/7-amino-actinomycin D (7-AAD) staining was used to measure the cell death as previously described^33^. Briefly, 5 × 10^5^ cells were resuspended in 100 μL binding buffer, and stained with 2.5 μl PE-Annexin V and 2.5 μL 7-AAD for 15 min at 37 °C in the dark, followed with adding of 400 μL binding buffer. Cells were analyzed by using flow cytometry with the FACS cytometer (BD Biosciences, Eysins, Switzerland).

### miRNAs profiling

HK-2 cells were subjected to H/R induction or Erastin-induced ferroptosis, and then the aberrant miRNAs expressions were analyzed by miRNAs sequencing as previously described^34,35^. Briefly, the total RNA of the cells was extracted by using the TRIzol reagent (Invitrogen, CA, USA) to prepare the small RNA sequencing library by using the NEBNext Multiplex Small RNA Library Prep Set for Illumina (NEB, USA) according to the manufacturer’s instruction., the libraries were denatured as single-stranded DNA molecules, captured on Illumina flow cells, amplified in situ as clusters and finally sequenced for per the manufacturer’s instructions. After sequencing, the Solexa CHASTITY quantity filtered reads were harvested as Clean Reads. For data analysis, differentially expressed miRNA profiles between two groups were compared, and fold change and *P*-value were calculated and used to identify significant differentially expressed miRNAs, and hierarchical clustering was performed. The intersection between the H/R- and ferroptosis-induced miRNA expression was conducted by the Venn diagram, and the selected miRNAs were verified the change of the expression by qRT-PCR.

### qRT-PCR analysis

Total RNA from tissues or cells was extracted using the TRIzol reagent (Invitrogen, CA, USA). Extracted RNA was reversely transcribed to cDNA using a PrimeScript RT reagent kit with gDNA Eraser (Takara, Tokyo, Japan), and real-time PCR was performed using SYBR Premix Ex TaqII (Takara), with U6 and GAPDH served as an inner control. The PCR condition was as follows: pre-denaturation at 95 °C for 15 min, followed by 40 cycles of 20 s of denaturation at 95 °C, an annealing at 55 °C for 30 s and an extension at 70 °C for 30 s. The fluorescence signals were collected at 72–95 °C after reaction for melting curve analysis. The results were analyzed using a hyperbolic curve and the relative gene expression was determined. The forward primer sequences of miR-182-5p were F: 5′-ATCACTTTTGGCAATGGTAGAACT-3′; R: 5′-TATGGTTTTGACGACTGTGTGAT-3′. Primer sequences for miR-378a-3p were F: 5′-GCGCACTGGACTTGGAGTC-3′; R: 5′-GCAGGGTCCGAGGTATTC-3′. Primer sequences for GPX4 were F: 5′-ATACGCTGAGTGTGGTTTGC-3′; R: 5′-CTTCATCCACTTCCACAGCG-3′. Primer sequences for SLC7A11 was F: 5′-ATACGCTGAGTGTGGTTTGC-3′; R: 5′-CTTCATCCACTTCCACAGCG-3′. Primer sequences for U6 were F: 5′-GCTTCGGCAGCACATATACTAA-3′; R: 5′-AACGCTTCACGAATTTGCGT-3′. Primer sequences for GAPDH were F: 5′-TGTGTCCGTCGTGGATCTGA-3′; R: 5′-CCTGCTTCACCACCTTCTTGA-3′.

### TUNEL assay

For TUNEL assay in tissues, the assay was performed in 3-mm-thick sections of paraffin-embedded tissue with the TUNEL assay Kit (Beyotime, Shanghai, China) according to the manufacturer’s instructions. Briefly, the paraffin-embedded tissue sections were dewaxed in dimethylbenzene and ethanol successively. Then the sections were treated with Protein Kinase K (Beyotime, Shanghai, China) for 30 min. After endogenous peroxidase was deactivated by using 3% H_2_O_2_, the sections were incubated with the biotin-dUTP buffer for 60 min. The sections were observed after treated with Streptavidin-HRP solution for 30 min, and the DAB buffer (Beyotime, Shanghai, China) for 5 min. For TUNEL assay in cells, the cells were washed with PBS and then fixed in 4% paraformaldehyde for 30 min following the 5 min-treatment with 0.3% Trition X-100. Then the cells were incubated with the TUNEL detection buffer for 60 min. DAPI was used to stain the nucleus. The cells were observed the fluorescence intensity under the fluorescence microscope (Leica DM1000).

### Western blot analysis

Total protein of cells or tissues were extracted with RIPA lysate buffer (Beyotime, Shanghai, China). After measuring the protein concentrations by BCA protein assay, 40 μg of total protein was loaded and separated by using the 12% SDS-PAGE gels, following the transfer to polyvinylidene fluoride (PVDF) membranes (Merck Millipore, Billerica, MA, USA). After blocking the membrane with 5% skim milk, the membranes were incubated with the indicated primary antibodies overnight at 4 °C. Then the membranes were incubated with horseradish peroxidase-linked IgG secondary antibody (Bioworld, USA) at room temperature for 1 h. The protein band was exposed with ECL buffer following the intensity analysis with ImageJ software. The primary GPX4, Keap1, Nrf2, NOX1, COX2 antibody used in the studies was purchased from Abcam (Anti-GPX4, ab125066; Ant-Keap1, ab196346; Anti-Nrf2, ab31163; Anti-NOX1, ab55831; Anti-COX2, ab15191). ACSL4 antibody was purchased from Santa Cruz Biotechnology (Anti-ACSL4, sc-365230). FTH1 antibody was purchased from Cell signaling Technology (Anti-FTH1, #3998S). SLC7A11 polyclonal antibody was purchased from Invitrogen (Anti-SLC7A11, PA5-18599). GAPDH (ab9485, Abcam, USA) was served as a loading control.

### Dual-luciferase reporter assay

Luciferase reporter assay was used to confirm the relationship between the miRNAs and the target genes. The sequences of 3′UTR of GPX4 and SLC7A11 mRNA were chemically synthesized and introduced into the luciferase reporter vector to constructed the wild-type (WT) luciferase reporter plasmids, respectively. The seed regions of miR-182-5p in the 3′-UTR of GPX4 and the seed regions of miR-378a-3p in the 3′-UTR of SLC7A11 were mutated to constructed mutant (Mut) luciferase reporter plasmids. The co-transfections with luciferase reporter plasmids and miRNA mimics or inhibitors to TCMK-1 cells and HK-2 cells were performed after the cells reaching 80% confluence by using Lipofectamine 3000 (Life Technologies, CA, USA) according to the manufacturer’s instruction. Twenty-four hours later, the cells were collected and dual-luciferase activity was measured using the Dual-Luciferase Reporter Assay (Promega, Shanghai, China) according to the manufacturer’s instructions and normalized to Renilla signals.

### RIP assay

The RIP experiment was conducted using the EZ-Magnetic RIP kit (Millipore, MA, USA) according to the manufacturer’s instructions. In brief, HK-2 and TCMK-1 cells at 80% confluency were lysed in complete RIP lysis buffer (~1 × 10^7^ cells/150 μL), following with immunoprecipitation with RIP buffer including magnetic beads coupled with anti-Ago2 antibody (Abcam) at room temperature for 4 h. Anti-IgG was used as a negative control. After the immobilized magnetic beads bounded complexes with magnet, the unbound materials were washed off. Then samples were incubated with Proteinase K to purified the RNA, and the immunoprecipitated RNA was isolated. qRT-PCR was performed to analyze miR-182-5p, miR-378a-3p, GPX4, and SLC7A11 level in the precipitates.

### Fluorescence in situ hybridization

The fluorescence in situ hybridization (FISH) assay was performed in tissues to observe the intracellular localization of the miRNAs as previously described^36,37^. The renal tissues of the rats in Sham group and IR group were isolated and frozen and embed in the Tissue-Tek to cut into 10 μM cryostat sections. After fixed and acetylated, the sections were washed and subject to in situ hybridization. Dig-labeled miR-182-5p and miR-378a-3p probes were used in the hybridization. The signals of Dig-labeled miR-182-5p and miR-378a-3p probes were detected using a tyramide signal amplification (TSA) kit (Thermofisher, CA, USA). Nuclei were counterstained with DAPI. Images were acquired on a Leica DM1000 confocal microscope (Leica Microsystems, Mannheim, Germany).

### Renal I/R injury model and histopathological studies

Male Sprague-Dawley (SD) rats (5 weeks old, weighting 180–220 g) were purchased from Shanghai SLAC Laboratory Animal Co., Ltd. The experimental protocol in the present study was approved by the Institutional Animal Ethical and Use Committee of the Institutional Animal Ethical and Use Committee of the First Affiliated Hospital of Xi’an Jiaotong University. The renal I/R injury model was created as described previously^38,39^. Briefly, SD rats were anesthetized with an intraperitoneal (i.p.) injection of pentobarbital sodium (25 mg/kg) and placed on a surgical thermostator. Then the rats were subjected to an abdominal incision, and the right kidney was carefully liberated from surrounding tissue, and nephrectomy was performed. The left kidney was exposed after a midline incision, and the renal artery was clamped with non-traumatic clamps for 45 min, followed by restoring of the renal blood flow. The kidneys were harvested and the serum was collected 48 h after the surgery. The rats in sham group were subjected to an abdominal incision without clamping the renal artery. Downregulation of miRNAs was performed by tail injection with the related antagomirs (80 mg/kg) (Ribo bio, Shanghai, China) immediately after the surgery as previously described^40,41^. Ferrostatin-1 (Santa Cruz, CA, USA) was served as the ferroptosis inhibitor in AKI model by intraperitoneal injection at the concentration of 5 mg/kg. Serum creatinine (Cr) and blood urea nitrogen (BUN) were measured by using the Cr assay kit and BUN assay kit (Jiangcheng Bioengineering Institute, Nanjing, China) to evaluate the model constructed. For histopathological studies, kidney tissues were separated from the rats and were dewaxed and hydrated. After washed by water, the tissue slices were stained in hematoxylin solution for 5 min. Next, after differentiated by 1% hydrochloric alcohol for 15 s, the slices were washed with water. And then the tissue slices were stained by eosin solution for 1 min. The tissue slices were observed under an optical microscope (Olympus, Tokyo, Japan). Renal injury was graded using a 5-point scale: 0, normal kidney; 1, <10% necrosis; 2, 10%–25%; 3, 25%–75%; and 4, >75%, in a blinded fashion by two experimenters as previously described^42^.

### Statistical analysis

All values are presented as the mean ± S.E.M. of three independent experiments. M. Student’s *t*-test was used for two-group comparisons. Comparisons of parameters among three or more groups were performed by using one-way ANOVA followed by Bonferroni test for selected pairs with the use of GraphPad Prism 8 statistical software. *P* < 0.05 was considered statistically significant.

## Results

### MiR-182-5p and miR-378a-3p aberrantly expressed in the cells suffering from H/R-induced injury and ferroptosis

**Fig. 1:**
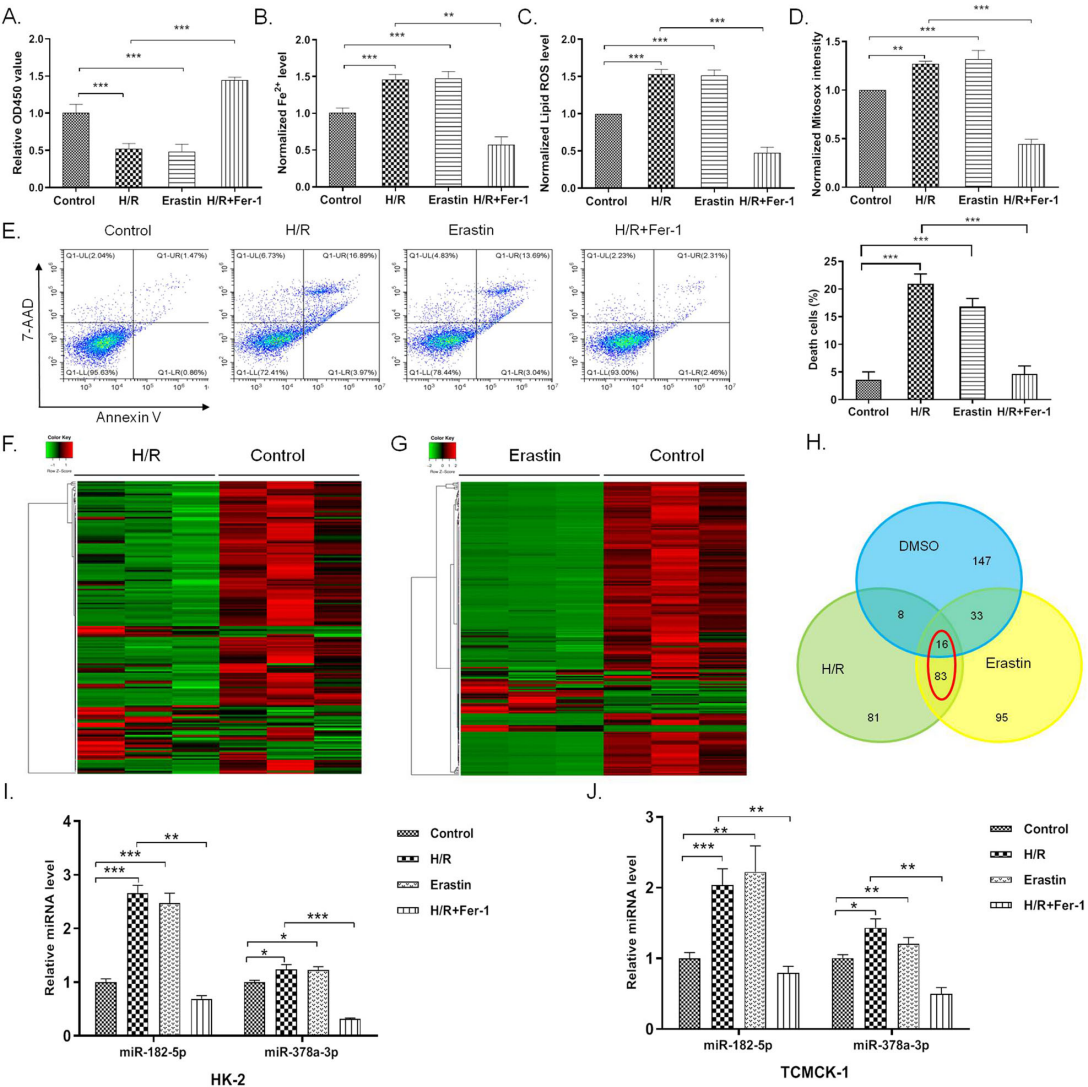
MiR-182-5p and miR-378a-3p aberrantly expressed in the cells suffering from H/R-induced injury and ferroptosis. HK-2 cells were treated with 1 μM ferrostatin-1 (Fer-1), 10 μM erastin or not 1 h before the H/R induction. **A** Fer-1 inhibited the decrease of cell viability induced by H/R. Fer-1 inhibited the increase of iron level induced by H/R. **C** Fer-1 inhibited the increase of Lipid ROS level induced by H/R. **D** Fer-1 inhibited the increase of mitochondrial superoxide level induced by H/R. **E** Fer-1 inhibited H/R-induced cell death. **F** Heat map showing expression profiles for the H/R-induced and G erastin-treated cells compared with the control group. **H** Venn diagram showed the intersection of the aberrantly expressed miRNAs in H/R-induced and erastin-treated cells. **I**, **J** Upregulation of miR-182-5p and miR-378a-3p in the H/R-induced and erastin-treated HK-2 and TCMK-1 cells were verified by qRT-PCR. *n* = 6. Data are presented as the mean ± s.e.m. of three independent experiments. **P < 0.01, ***P < 0.001 vs. indicated group.

To investigate whether ferroptosis functions in the process of H/R-induced renal epithelial cell injury, we treated the H/R-induced cells with ferroptosis inhibitor ferrostatin-1 (Fer-1) to observe if inhibition of ferroptosis could attenuate H/R-induced injury. As shown in Fig. 1A, inhibition of ferroptosis inhibited H/R-induced injury in HK-2 cells. Besides, H/R induced increased iron accumulation and lipid ROS level as ferroptosis inducer erastin did (Fig. 1B, C). Increased level of mitochondrial superoxide was obvious in both H/R-induced cells and erastin-treated cells as indicated by mitosox intensity (Fig. 1D). Treatment of ferroptosis inhibitor Fer-1 could abrogate the ferroptosis-related change in H/R-induced cells (Fig. 1B–D). H/R induction and erastin treatment induced cell death of HK-2 cells, while Fer-1 inhibited H/R-induced cell death, as indicated by the Annexin V/7-AAD assay (Fig. 1E). All these data suggested that H/R induced ferroptosis in the HK-2 cells, and inhibition of ferroptosis could attenuate H/R-induced injury in vitro.

Erastin, a reported inducer of ferroptosis^43^, showed the typical ferroptosis phenotype in HK-2 cells as the GPX4 inhibitor RSL3 did (Supplementary Fig. 1). To further investigate whether microRNA functioned in the regulation of ferroptosis in H/R-induced injury, we performed miRNAs profiling to screen the miRNAs that expressed aberrantly in the process of H/R-induced injury and erastin-induced ferroptosis (Fig. 1F, G). We found 83 miRNAs which showed the identical change in the process of H/R-induced injury and erastin-induced ferroptosis (Fig. 1H). Among the miRNAs, we identified that miR-182-5p and miR-378a-3p increased significantly in the H/R-induced and erastin-treated HK-2 cells, while inhibition of ferroptosis decreased the two miRNAs level in H/R-induced HK-2 cells (Fig. 1I). The same trend was also observed in TCMK-1 cells (Fig. 1J), indicating that miR-182-5p and miR-378a-3p may participate in the regulation of ferroptosis in H/R-induced renal cells injury.

### MiR-182-5p and miR-378-3p promotes ferroptosis in the renal epithelial cells

**Fig. 2:**
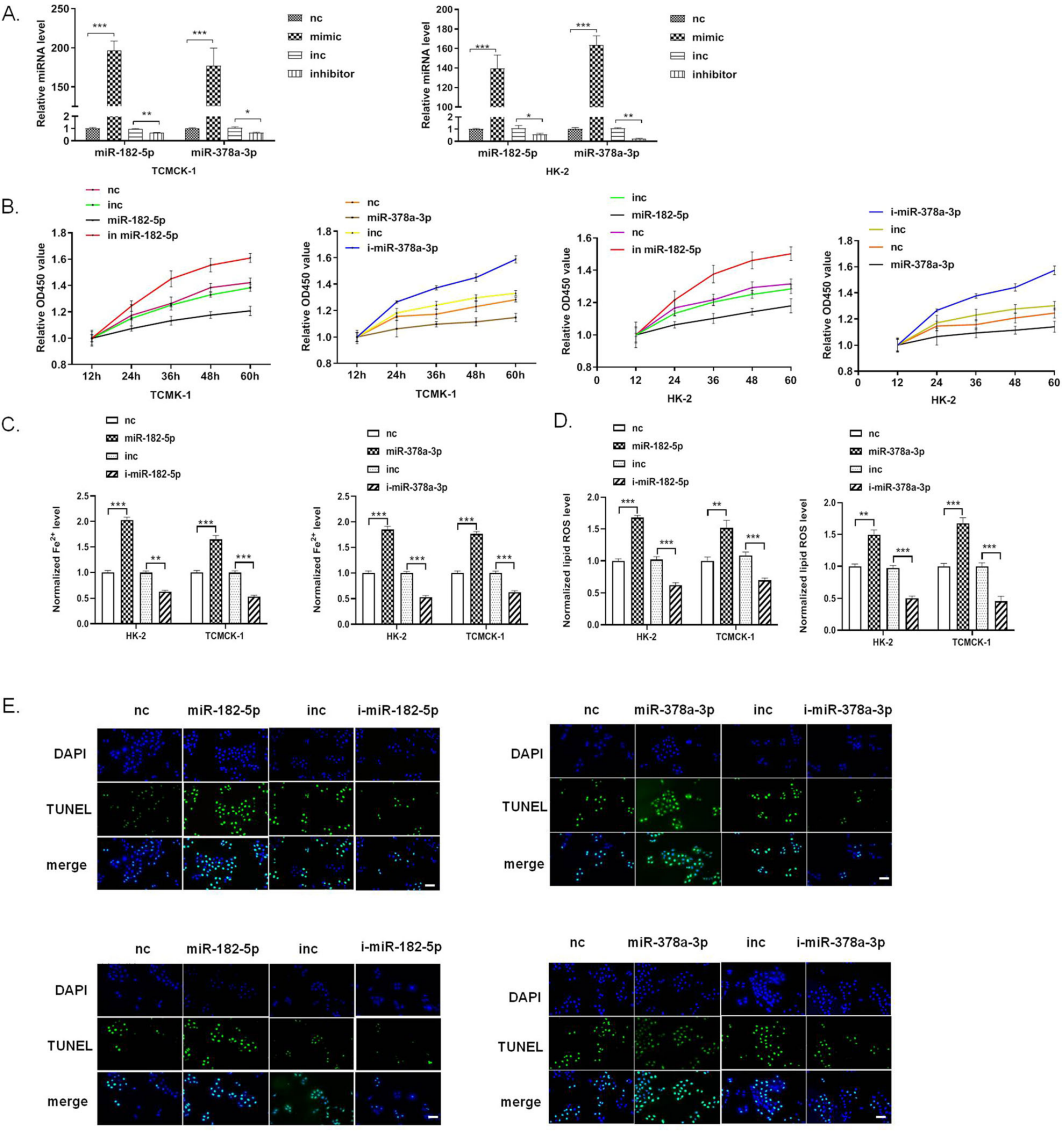
MiR-182-5p and miR-378-3p promotes ferroptosis in the renal epithelial cells. HK-2 and TCMK-1 cells were treated with miR-182-5p mimics (10 pmol) or inhibitor (40 pmol), or miR-378a-3p mimics (10 pmol) or inhibitor (40 pmol) for 36 h, then the cells were subjected to various detection. **A** The efficacy of the miRNAs mimics and inhibitors in TCMK-1 and HK-2 cells was verified by qRT-PCR. **B** Cell viability of TCMK-1 and HK-2 cells were reduced by miR-182-5p and miR-378a-3p mimics, and enhanced by miR-182-5p and miR-378a-3p inhibitors. **C** Iron level and **D** lip ROS level of TCMK-1 and HK-2 cells were elevated by miR-182-5p and miR-378a-3p mimics, and decreased by miR-182-5p and miR-378a-3p inhibitors. E miR-182-5p and miR-378a-3p mimics induced increasing of cell death of TCMK-1 and HK-2 cells, while miR-182-5p and miR-378a-3p inhibitors decreased the cell death, as indicated by TUNEL staining (green), Bar = 100 μm. *n* = 6. Data are presented as the mean ± s.e.m. of three independent experiments. *P < 0.05, **P < 0.01, ***P < 0.001 vs. indicated group.

We then performed a series of studies to investigate the roles of miR-182-5p and miR-378-3p in ferroptosis by using the miRNA mimics or inhibitor to upregulate or downregulate the miRNAs. The efficacy of the miRNA mimics or inhibitor in HK-2 and TCMK-1 cells was verified by qRT-PCR (Fig. 2A). We determined the effect of the two miRNAs on the HK-2 and TCMK-1 cell viability by using CCK-8 assay. As shown in Fig. 2B, miR-182-5p and miR-378-3p mimics significantly decreased the cell viability of HK-2 and TCMK-1 cells, while miR-182-5p and miR-378-3p inhibitor increased the cell viability. Iron accumulation increased obviously in miR-182-5p and miR-378-3p mimics-treated HK-2 and TCMK-1 cells, and decreased in miR-182-5p and miR-378-3p inhibitor-treated cells (Fig. 2C). The same trend of the ROS was also observed as the iron accumulation in each group (Fig. 2D). TUNEL assay showed that miR-182-5p and miR-378-3p mimics significantly induced cell death in HK-2 and TCMK-1 cells, and miR-182-5p and miR-378-3p inhibitor showed the totally opposite effect (Fig. 2E). miR-182-5p and miR-378-3p induced obvious cell death in HK-2 cells (Supplementary Fig. 2). All these data suggested that MiR-182-5p and miR-378-3p promote ferroptosis in the renal epithelial cells.

### MiR-182-5p and miR-378-3p promotes ferroptosis in the kidney of I/R injured rats

**Fig. 3:**
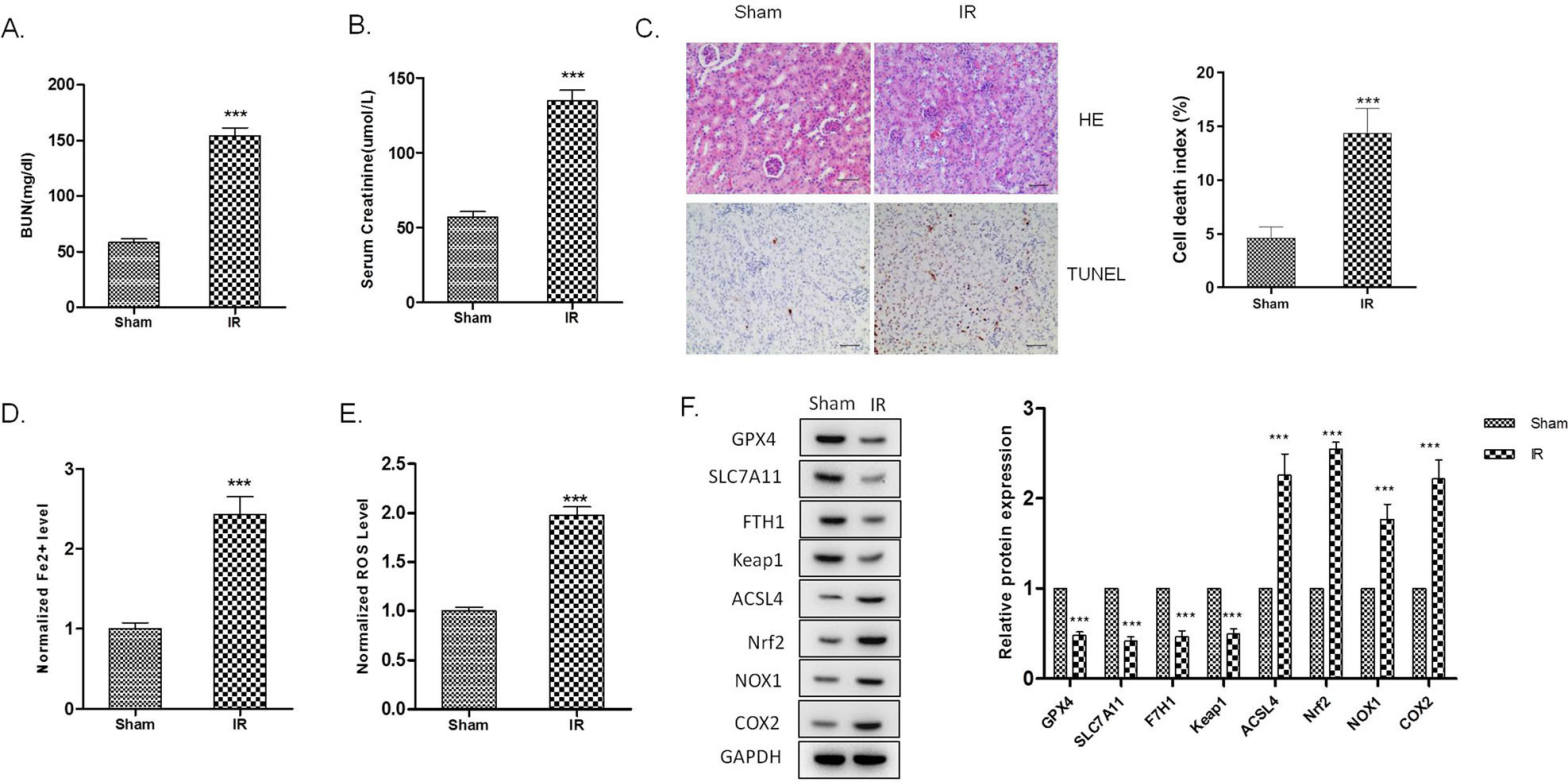
Ferroptosis is enhanced in the kidney of I/R injured rats. Rats renal artery was clamped with non-traumatic clamps for 45 min, followed by restoring of the renal blood flow. Control rats underwent sham surgery (sham group) without ischemia-reperfusion injury (IRI). Blood samples and renal tissues were collected 48 h after the injury. **A** Serum creatinine and **B** blood urea nitrogen levels were significantly higher in the I/R group than in the sham group. **C** Kidney tissue sections were subjected to histological examination by hematoxylin and eosin staining (H&E) and TUNEL assay to evaluate renal tubule injury. **D** Increased iron level and **E** ROS level in I/R renal tissues indicates the increase of ferroptosis. **F** Western blot was used to detect the indicated protein expression. The protein intensity was analyzed by using ImageJ. *n* = 6. Data are presented as the mean ± s.e.m. of three independent experiments. ***P < 0.001 vs. the sham group.

We further investigated the role of ferroptosis in the I/R kidney injury in vivo. We constructed the I/R-induced kidney injury in rats. Renal function was evaluated by detection of serum creatinine and BUN levels. Serum creatinine and BUN levels of the rats in I/R group increased significantly, as shown in Fig. 3A, B. The pathological condition of the I/R rats were evaluated by H&E staining and TUNEL assay, which manifested the increased injury of kidney tissues and cell death of the renal epithelial cells in I/R rats (Fig. 3C). In the renal tissues of I/R rats, iron accumulation and ROS level elevated significantly, indicating the enhancement of ferroptosis in I/R-induced renal injury (Fig. 3D, E). A series of gene expression involved in the progress of ferroptosis, including the downregulation of GPX4, SLC7A11, FTH1, and the upregulation of ACSL4, NOX1, and COX2^44^. We found that the GPX4, SLC7A11, FTH1, and Keap1 were downregulated, and Nrf2, NOX1, and COX2 were upregulated in the I/R rats’ kidney tissues, indicating the activation of ferroptosis in the I/R-induced kidney injury (Fig. 3F).

**Fig. 4:**
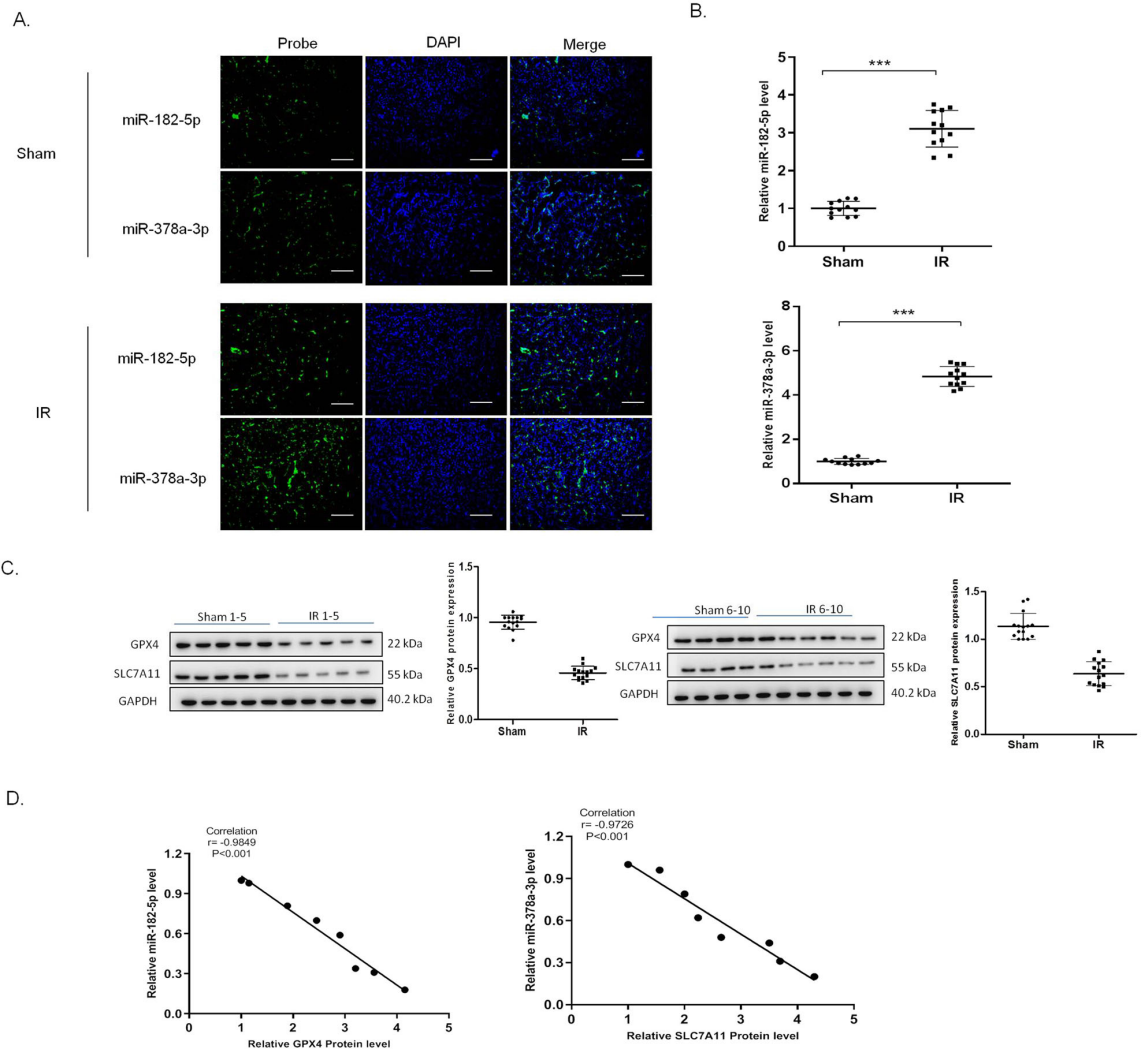
MiR-182-5p and miR-378a-3p negatively correlates with the GPX4 and SLC7A11 expression, respectively. **A** The representative photograph showing fluorescence in situ hybridization in renal tissues, green represented biotin-labeled probe against miR-182-5p and miR-378a-3p and blue represents DAPI staining of the nucleus. **B** miR-182-5p and miR-378a-3p expression increased in the I/R renal injury rats. **C** Western blot was used to detect the GPX4 and SLC7A11 protein expression in the renal tissues. The protein intensity was analyzed by using ImageJ. **D** Pearson analysis showed the negative correlation of miR-182-5p and miR-378a-3p with GPX4 and SLC7A11 protein expression. *n* = 6. Data are presented as the mean ± s.e.m. of three independent experiments. ***P < 0.001 vs. the sham group.

Further analysis by FISH assay revealed that miR-182-5p and miR-378a-3p increased in the nucleus area of the renal tissues in the I/R group (Fig. 4A). qRT-PCR showed that miR-182-5p and miR-378a-3p expression increased in the I/R group (Fig. 4B). The protein expression of GPX4 and SLC7A11 was shown in Fig. 4C, in which the expression of GPX4 and SLC7A11 decreased significantly in the I/R group. Moreover, we found the negative correlation between miR-182-5p and the GPX4 expression, and the negative correlation between miR-378a-3p with SLC7A11 expression by using Pearson Correlation analysis (Fig. 4D).

### MiR-182-5p targets GPX4 and miR-378a-3p targets SLC7A11, respectively, in the renal epithelial cells

**Fig. 5:**
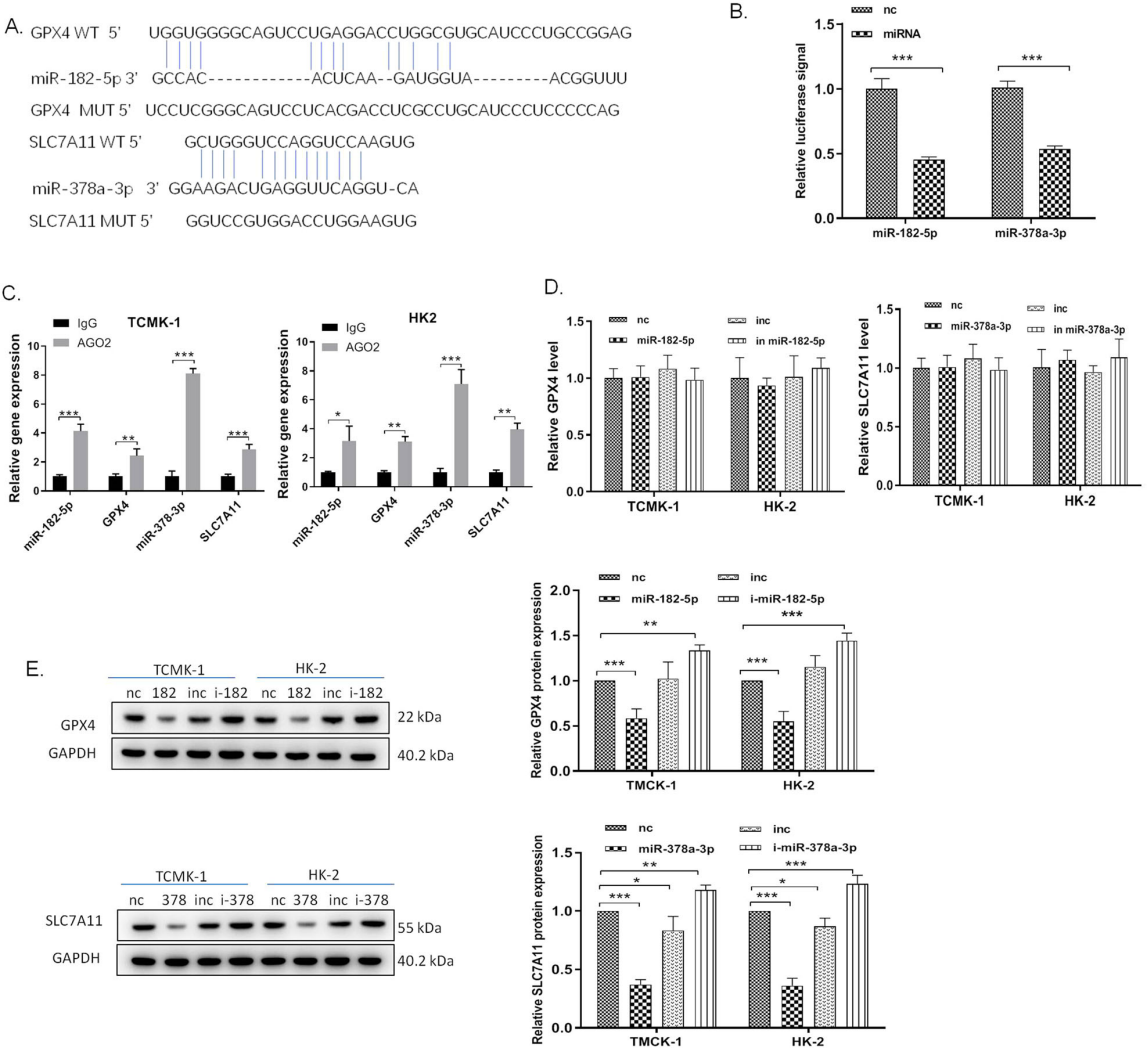
MiR-182-5p targets GPX4 and miR-378a-3p targets SLC7A11, respectively, in the renal epithelial cells. **A** The predicted binding site between 3′UTR of GPX4 mRNA and miR-182-5p, and the predicted binding site between 3′UTR of SLC7A11 mRNA and miR-378a-3p. **B** HK-2 cells were co‑transfected with luciferase constructs containing the GPX4 WT or MUT 3′‑UTRs and miR‑182-5p mimics or mimics NC, or co‑transfected with luciferase constructs containing the SLC7A11 WT or MUT 3′‑UTRs and miR‑378a-3p mimics or mimics NC. Luciferase activity was measured. **C** RIP assay followed by qRT-PCR to assay miR-182-5p and miR-378a-3p endogenously associated with GPX4 and SLC7A11, respectively. **D** miR-182-5p and miR-378a-3p scarcely influence the GPX4 and SLC7A11 mRNA levels. **E** The GPX4 and SLC7A11 expression levels were downregulated after treated with miR-182-5p and miR-378a-3p mimics and upregulated with the treatment with miRNAs inhibitors in HK-2 and TCMK-1 cells. The protein intensity was analyzed by using ImageJ. *n* = 6. Data are presented as the mean ± s.e.m. of three independent experiments. *P < 0.05, **P < 0.01, ***P < 0.001 vs. indicated group.

To further explore the mechanism of miR-182-5p and miR-378a-3p’s regulatory effect, we performed bioinformatics assay to predict the potential targeting gene of the miRNAs by using TargetScan. We found that GPX4 and SLC7A11 are the potential target gene of miR-182-5p and miR-378a-3p, respectively (Fig. 5A). Luciferase reporter assay was used to verify the direct targeting effect of miR-182-5p to GPX4 and miR-378a-3p to SLC7A11. We transfected miR-182-5p mimics or miR-378a-3p mimics with the wild-type GPX4 or SLC7A11 luciferase reporter vector into HK-2 cells and detected the luciferase activity. As shown in Fig. 5B, co-transfection of miR-182-5p with wild-type GPX4 luciferase reporter caused a sharp decrease of luciferase activity compared with the mutant GPX4 luciferase reporter, and transfection of miR-378a-3p decreased the luciferase activity in the cells which were simultaneously transfected with wild-type SLC7A11 luciferase reporter, indicating that miR-182-5p and miR-378a-3p directly bound to the 3′UTR of GPX4 and 3′UTR of SLC7A11, respectively. Moreover, RIP assay further showed that GPX4 mRNA-miR-182-5p, and SLC7A11 mRNA-miR-378a-3p were specifically enriched in the component pulled down by anti-Ago2 antibody in TMCK-1 and HK-2 cells (Fig. 5C), indicating that GPX4 and SCL7A11 mRNA was associated with RNA-induced silencing complex composed of miR-182-5p and miR-378a-3p. Results from qPCR and western blot further showed that miR-182-5p and miR-378a-3p negatively regulated the expression of GPX4 and SCL7A11, but had no obvious effect on their mRNA level (Fig. 5D, E).

### MiR-182-5p and miR-378a-3p regulates ferroptosis in the renal epithelial cells by targeting GPX4 and SLC7A11, respectively

**Fig. 6:**
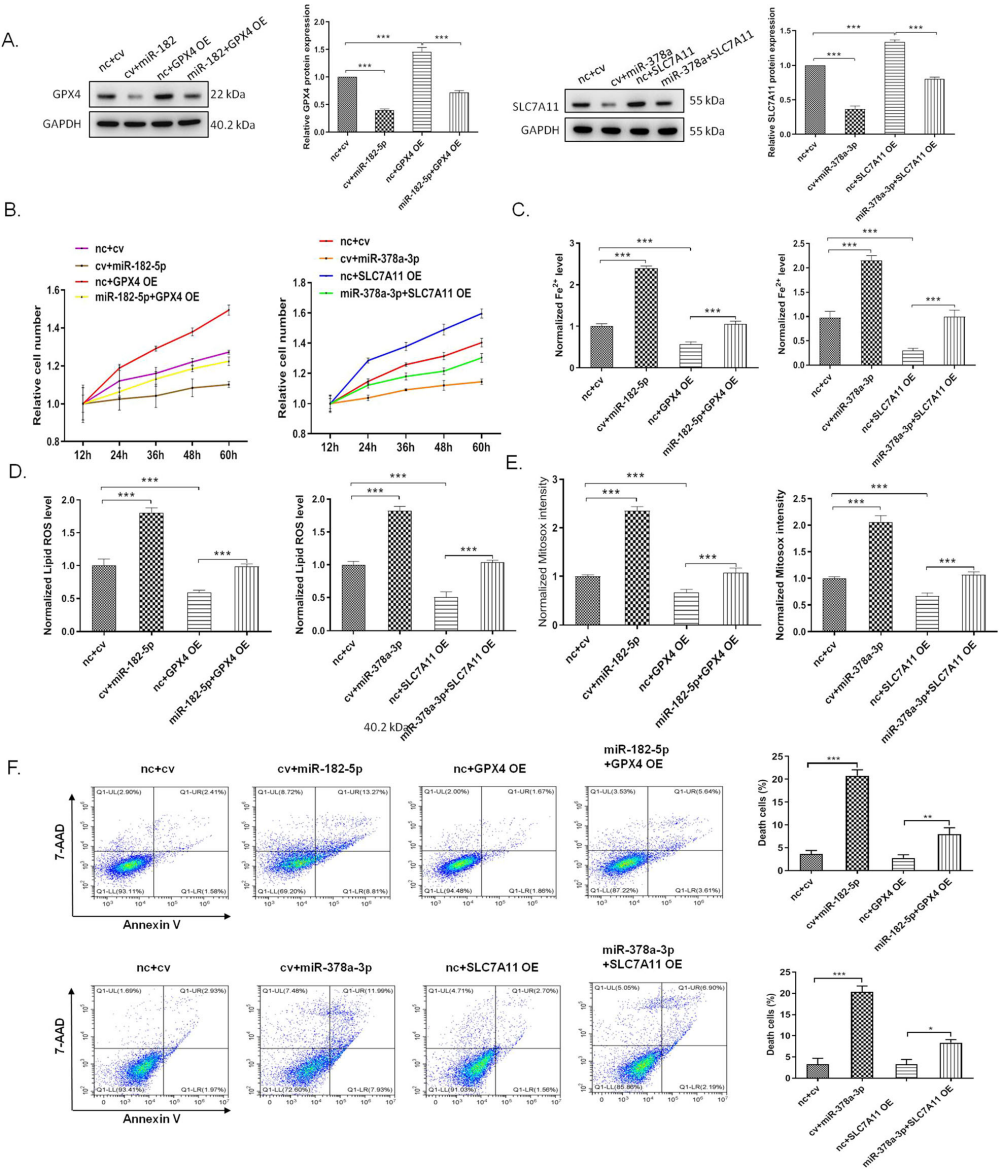
MiR-182-5p and miR-378a-3p regulates ferroptosis in the renal epithelial cells by targeting GPX4 and SLC7A11, respectively. The HK-2 cells were treated with indicated miRNA mimic (10 pmol) and GPX4 overexpression lentivirus (MOI = 50) with a final concentration of 5 μg/mL polybrene for, or co-treated with the miRNA mimic and lentivirus for 36 h, then the cells were subjected to various detection. nc, control mimic; cv, control lentivirus. **A** miR-182-5p and miR-378a-3p inhibited the GPX4 and SLC7A11 protein expression which were upregulated by the lentivirus treatment. **B** Overexpression of GPX4 and SLC7A11 inhibited the reduction of cell viability induced by miRNA mimic. **C** Overexpression of GPX4 and SLC7A11 inhibited the increase of cell iron level, ROS level (**D**), and mitosox intensity (**E**) induced by miRNA mimic. **F** Overexpression of GPX4 and SLC7A11 inhibited the miRNA mimic-induced cell death of HK-2 cells. *n* = 6. Data are presented as the mean ± s.e.m. of three independent experiments. **P < 0.01, ***P < 0.001 vs. indicated group.

To evaluate whether miR-182-5p and miR-378a-3p regulated ferroptosis in the renal epithelial cells by targeting GPX4 and SLC7A11, we treated the cells with miR-182-5p (or miR-378a-3p), GPX4 lentivirus (or SLC7A11 lentivirus), and the co-treatment of miRNA mimic and the overexpression lentivirus. Western blot showed that treatment with overexpression lentivirus could inhibit the miRNAs-induced downregulation of the protein expression (Fig. 6A). Overexpression of GPX4 and SLC7A11 increased the cell viability which was decreased by miR-182-5p and miR-378a-3p (Fig. 6B). For the regulation of ferroptosis, iron accumulation, lipid ROS level, and mitosox intensity were detected. As shown in Fig. 6C, miR-182-5p and miR-378a-3p increased the iron level in cells which could be reversed by overexpression of GPX4 and SLC7A11. miR-182-5p and miR-378a-3p increased the lipid ROS level and mitochondrial superoxide in cells, while overexpression of GPX4 and SLC7A11 abolished the effect of miR-182-5p and miR-378a-3p (Fig. 6D, E). Further cell death analysis revealed that overexpression of GPX4 and SLC7A11 inhibited the HK-2 cell death induced by miR-182-5p and miR-378a-3p (Fig. 6F). All these data suggested that miR-182-5p and miR-378a-3p regulate ferroptosis in the renal epithelial cells by targeting GPX4 and SLC7A11, respectively.

### Inhibition of miR-182-5p and miR-378a-3p attenuates I/R-induced kidney injury in vivo

**Fig. 7:**
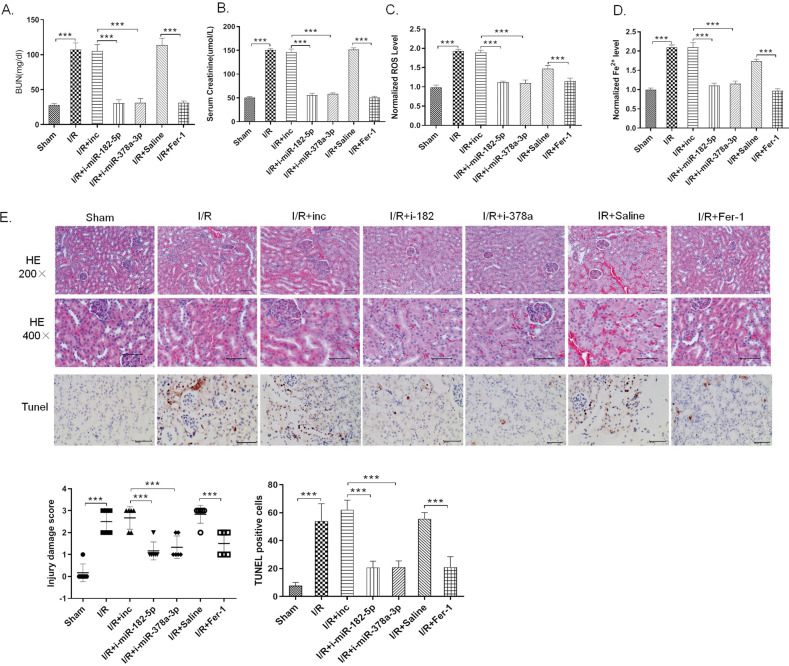
Inhibition of miR-182-5p and miR-378a-3p attenuates I/R-induced kidney injury in vivo. The rats were divided into seven groups: Sham group, I/R model group, I/R + nc-antagomir group (I/R + inc), I/R + miR-182-5p antagomir group (I/R + i-miR-182-5p), I/R + miR-378a-3p antagomir group (I/R + i-miR-378a-3p), I/R + saline group (I/R + saline), and I/R + Ferostatin-1 group (I/R + Fer-1), with 10 rats in each group. Antagomir was administrated by tail injection at the dosage of 80 mg/kg and Fer-1 was served as the ferroptosis inhibitor in AKI model by intraperitoneal injection at the concentration of 5 mg/kg. **A** Serum creatinine and **B** blood urea nitrogen levels were significantly decreased in the antagomir and Fer-1 administrated group than in the I/R group. **C** Iron level and **D** ROS level decreased in the antagomir and Fer-1 administrated group than in the I/R group. **E** H&E and TUNEL assay showed reduced renal tubule injury in the antagomir and Fer-1 administrated group than in the I/R group. *n* = 10. Data are presented as the mean ± s.e.m. **P < 0.01, ***P < 0.001 vs. indicated group.

Antagomirs are a novel class of chemically engineered oligonucleotides which could silence endogenous miRNAs in vivo efficiently and specifically^45^. In the present studies, we used the indicated antagomirs to silence the miRNAs in rats to see if downregulation of miR-182-5p and miR-378a-3p would show benefits in I/R rats. The rats were divided into seven groups: Sham group, I/R model group, I/R + nc-antagomir group (I/R + inc), I/R + miR-182-5p antagomir group (I/R + i-miR-182-5p), I/R + miR-378a-3p antagomir group (I/R + i-miR-378a-3p), I/R + saline group (I/R + saline), and I/R + Ferostatin-1 group (I/R + Fer-1), with 10 rats in each group. Antagomir was administrated by tail injection at the dosage of 80 mg/kg. Fer-1 was served as the ferroptosis inhibitor in AKI model by intraperitoneal injection at the concentration of 5 mg/kg. As shown in Fig. 7A, B, antagomir of miR-182-5p and miR-378a-3p inhibited the I/R-induced increase of serum creatinine and BUN level, as Fer-1 does. Antagomir of miR-182-5p and miR-378a-3p as well as Fer-1 inhibited the I/R-induced pathological change and cell death in the renal tissues (Fig. 7E). I/R induced the increase of ROS and iron level in the renal tissues, which could be apparently inhibited by antagomir of miR-182-5p and miR-378a-3p as well as Fer-1, indicating their abilities to attenuate the ferroptosis in I/R kidney injury (Fig. 7C, D).

## Discussion

Ischemia/reperfusion renal injury is the leading cause of ARF, a disease with high morbidity and mortality because of the lack of effective treatment^9,38^. Renal tubular cell death induced by I/R was considered as the main cause of ARF^19,46^. In the present study, we confirmed the occurrence of I/R-induced ferroptosis in vivo and in vitro. Further study demonstrated that I/R-induced upregulation of miR-182-5p and miR-378a-3p to downregulate the expression of GPX4 and SLC7A11, which collectively contributed to the process of ferroptosis.

Ferroptosis is a distinct form of RCD characterized by increased ROS level and lipid peroxidation and iron accumulation, which has been found in multiple cell-death related disease^47–49^. In an in vitro model of acute injury in renal tubular cells, inhibition of ferroptosis protected the cells from the injury^50^. In the present study, we established the I/R-induced kidney injury model in vivo, and used H/R-induced injury model in vitro to mimic the I/R injury. We found that H/R induced increased levels of Fe^2+^ and ROS in cells as the ferroptosis inducer erastin does, while inhibition of ferroptosis by using Fer-1 reduced H/R-induced cell injury. Consistently, in vivo studies showed that I/R induced increased levels of Fe2+ and ROS in kidney tissues, along with the change of the expression of ferroptosis-related genes, suggesting that ferroptosis played important roles in the I/R-induced renal injury.

We further performed miRNA profile to screen the miRNAs that may regulate H/R-induced injury and ferroptosis, and identified that miR-182-5p and miR-378a-3p expressed aberrantly in H/R-induced and erastin-induced cells. In vivo studies showed that miR-182-5p and miR-378a-3p increased in the nucleus area of the renal tissues in the I/R group, indicating that they may regulate gene expression during the process of ferroptosis in I/R injury. It was reported that miR-182-5p was oncogenic and it could promote cell proliferation^51^. However, its role may be controversial because inhibition of miR-182-5p ameliorated ischemic-induced acute kidney injury in rats^52^. Besides, miR-182-5p was significantly increased in posttransplantation AKI patients and showed statistical correlations with global gene expression changes during the development of AKI^53^. In the present studies, we found that overexpression of miR-182 increased cell ferroptosis and cell death, approving the idea that miR-182-5p was harmful to the cell viability in the I/R injury. miR-378a-3p was reported to exhibit anti-proliferation effect in myoblasts^54^. MiR-378a-3p elevated significantly in the urine of patients with I/R-induced kidney injury^55^. Consistently, we found that miR-378a-3p was upregulated in the I/R rats’ kidney, promoted the ferroptosis in renal epithelial cells.

GPX4 is a negative regulator of ferroptosis because it could reduce lipid hydroperoxides^16^. In mice, knockout of GPX4 induced severe acute kidney failure, which could be rescued by Fer-1 treatment^56^. SLC7A11, also called xCT, is the main component of system x_c_-, which transports cysteine into cells to alleviate the cell peroxidation^57^. In I/R-triggered ARF mice, knockout of xCT aggravated the renal injury^58^. Interestingly, we found that miR-182-5p negatively correlated with GPX4 expression, and miR-378a-3p negatively correlated with SLC7A11 expression in the I/R rats, indicating that there may be some regulatory relationship among them. We further found that there are potential binding sites between the miR-182-5p and GPX4, as well as miR-378a-3p and SLC7A11 by performed bioinformatics analysis. Luciferase reporter assay, RIP assay, and western blot further proved that miR-182-5p and miR-378a-3p could downregulate the expression of GPX4 and SLC7A11 by directly targeting the 3′UTR of GPX4 and SLC7A11 mRNA, respectively. Overexpression of GPX4 or SLC7A11 could abrogate increasing ferroptosis induced by miR-182-5p and miR-378-3p mimics in cells. In vivo studies further showed that in vivo silencing miR-182-5p and miR-378-3p alleviated I/R-induced ferroptosis and kidney injury in rats, as the same effect as the Fer-1 treatment did.

In conclusion, we demonstrated that ferroptosis played important roles in the I/R-induced renal injury. I/R-induced upregulation of miR-182-5p and miR-378-3p to induce the activation of ferroptosis by directly targeting GPX4 and SLC7A11. The process of ferroptosis may be regulated collectively by multiple miRNAs induced by I/R.

## Supplementary information

Supplementary table1

Supplementary Figure Legends

Supplementary Figure1

Supplementary Figure2

Supplementary Figure3
