# Supplementary material for: miR-182-5p and miR-378a-3p regulate ferroptosis in I/R-induced renal injury
Source: Cell Death Dis. 2020 Oct 28;11(10):929. doi: 10.1038/s41419-020-03135-z (PMC7595188; doi:10.1038/s41419-020-03135-z)
Supplement: Supplementary file 1 — Supplementary table1 [file 41419_2020_3135_MOESM1_ESM.docx]

**Table S1. Primer sequences of genes in RT-qPCR assay**

| **Gene** | **Forward Primer** | **Reversed Primer** |
| --- | --- | --- |
| MiR-182-5p | ATCACTTTTGGCAATGGTAGAACT | TATGGTTTTGACGACTGTGTGAT |
| MiR-378a-3p | GCGCACTGGACTTGGAGTC | GCAGGGTCCGAGGTATTC |
| GPX4 | ATACGCTGAGTGTGGTTTGC | CTTCATCCACTTCCACAGCG |
| SLC7A11 | ATACGCTGAGTGTGGTTTGC | CTTCATCCACTTCCACAGCG |
| U6 | GCTTCGGCAGCACATATACTAA | AACGCTTCACGAATTTGCGT |
| GAPDH | TGTGTCCGTCGTGGATCTGA | CCTGCTTCACCACCTTCTTGA |
