# Supplementary material for: miR-182-5p and miR-378a-3p regulate ferroptosis in I/R-induced renal injury
Source: Cell Death Dis. 2020 Oct 28;11(10):929. doi: 10.1038/s41419-020-03135-z (PMC7595188; doi:10.1038/s41419-020-03135-z)

**Supplementary figure 1**

**Ferrostatin-1 inhibits ferroptosis induced by erastin and RSL3.**

HK-2 cells were treated with 1 μM ferrostatin-1 (Fer-1), 10 μM erastin or 1 μM RSL3. (A) Fer-1 inhibited the decrease of cell viability induced by erastin. (B) Fer-1 inhibited the increase of iron level induced by erastin. (C) Fer-1 inhibited the increase of Lipid ROS level induced by erastin. (D) Fer-1 inhibited the increase of mitochondrial superoxide level induced by erastin. (E) Fer-1 inhibited the erastin-induced depletion of GSH level. (F) Fer-1 inhibited the decrease of cell viability induced by RSL3. (B) Fer-1 inhibited the increase of iron level induced by RSL3. (C) Fer-1 inhibited the increase of Lipid ROS level induced by RSL3. (D) Fer-1 inhibited the increase of mitochondrial superoxide level induced by RSL3. (E) Fer-1 inhibited the RSL3-induced depletion of GSH level. n=6. Data are presented as the mean ± s.e.m. of three independent experiments. ##P<0.01, ###P<0.001, vs. Control group; **P<0.01, ***P<0.001 vs. Erastin or RSL3 group.


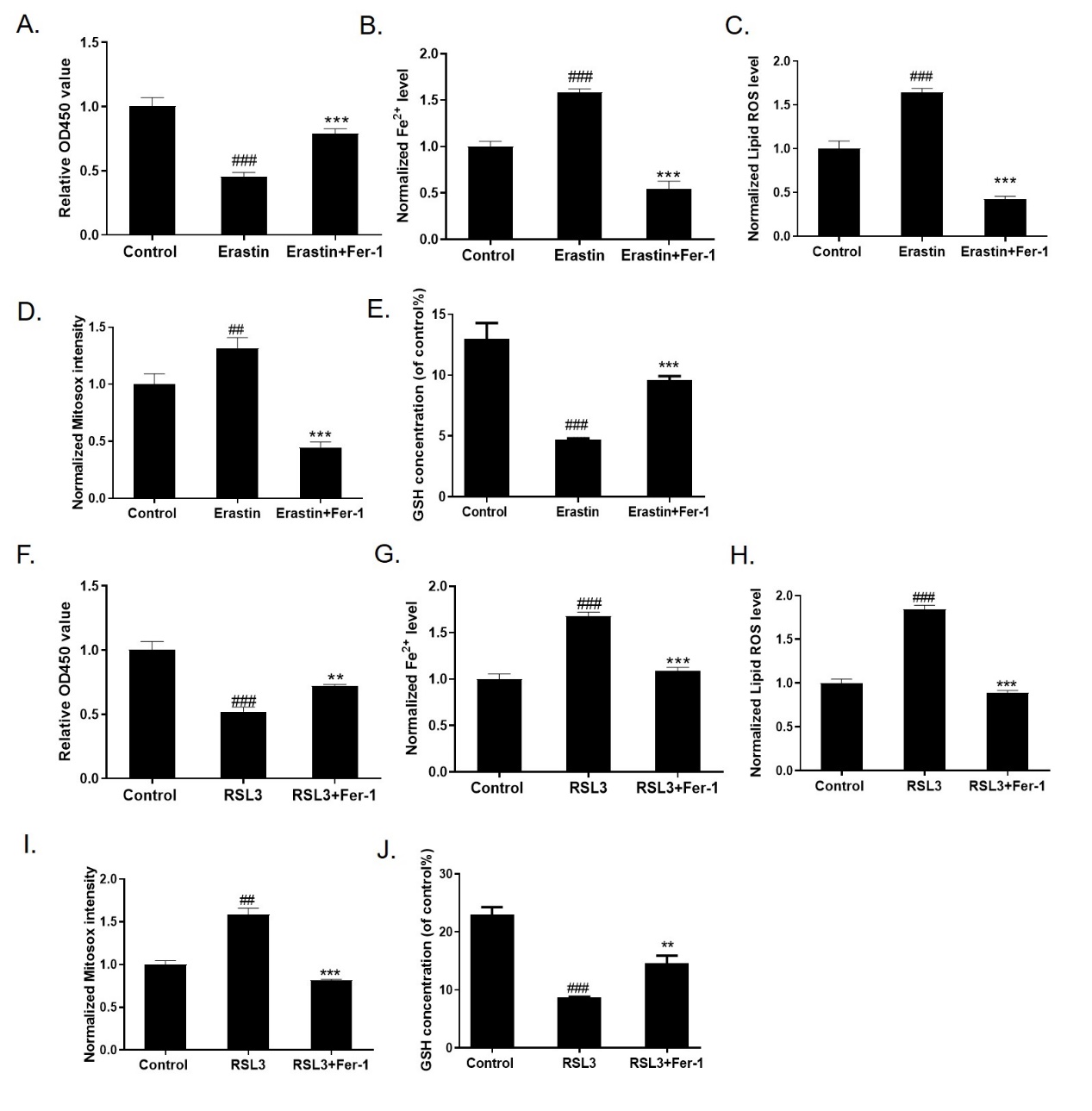


**Supplementary figure 2**

**MiR-182-5p and miR-378-3p promotes cell death in the renal epithelial cells.**

HK-2 cells were treated with miR-182-5p mimics (10 pmol) or inhibitor (40 pmol), or miR-378a-3p mimics (10 pmol) or inhibitor (40 pmol) for 36 h, then the cells were subjected to Annexin V/7-AAD staining followed with flow cytometry to measure the cell death. n=6. Data are presented as the mean ± s.e.m. of three independent experiments. *P<0.05, ***P<0.001 vs. indicated group.


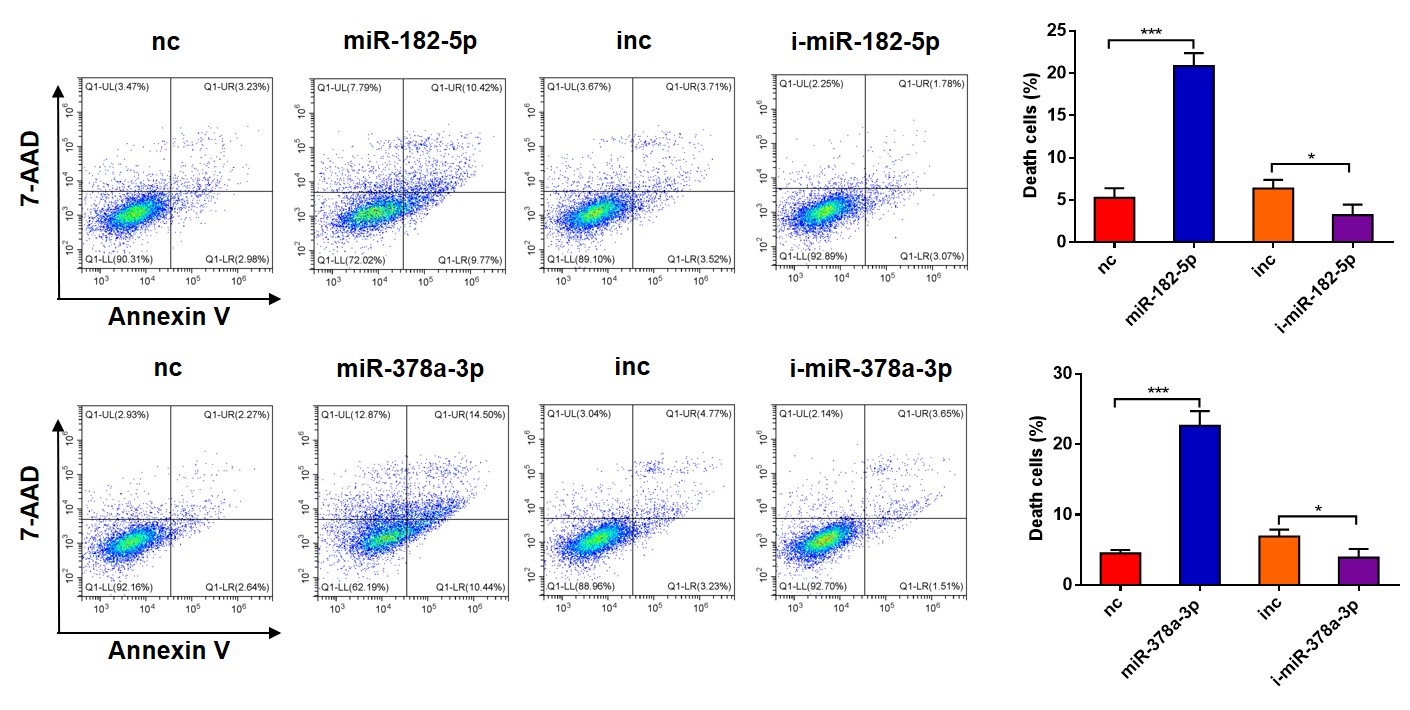


**Supplementary figure 3**

**MiR-182-5p and miR-378-3p-induced HK-2 cell death is independent of apoptosis and necrosis.**

HK-2 cells were treated with miR-182-5p mimics (10 pmol) or miR-378a-3p mimics (10 pmol) with or without apoptosis inhibitor Ac-DEVD-CHO (200 μM) or necrosis inhibitor necrosis inhibitor necrostatin-1 (50 μM) for 36 h, then the cells were subjected to Annexin V/7-AAD staining followed with flow cytometry to measure the cell death. n=6. Data are presented as the mean ± s.e.m. of three independent experiments. ***P<0.001 vs. NC group; ns, no significance.


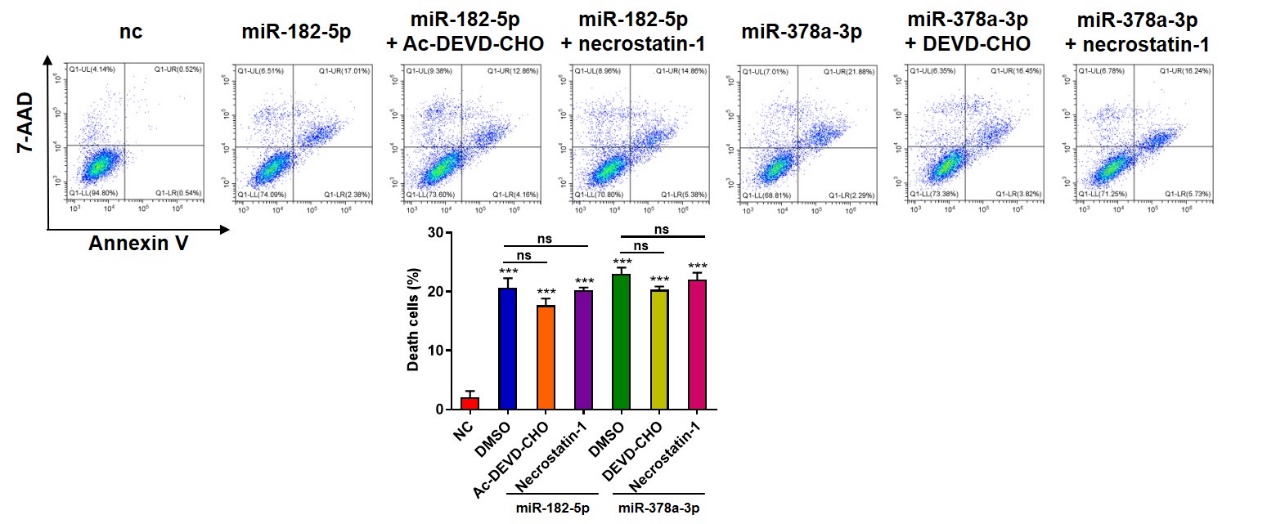

Supplement: Supplementary file 2 — Supplementary Figure Legends [file 41419_2020_3135_MOESM2_ESM.docx]
