# Supplementary figures and images for: miR-182-5p and miR-378a-3p regulate ferroptosis in I/R-induced renal injury
Source: Cell Death Dis. 2020 Oct 28;11(10):929. doi: 10.1038/s41419-020-03135-z (PMC7595188; doi:10.1038/s41419-020-03135-z)

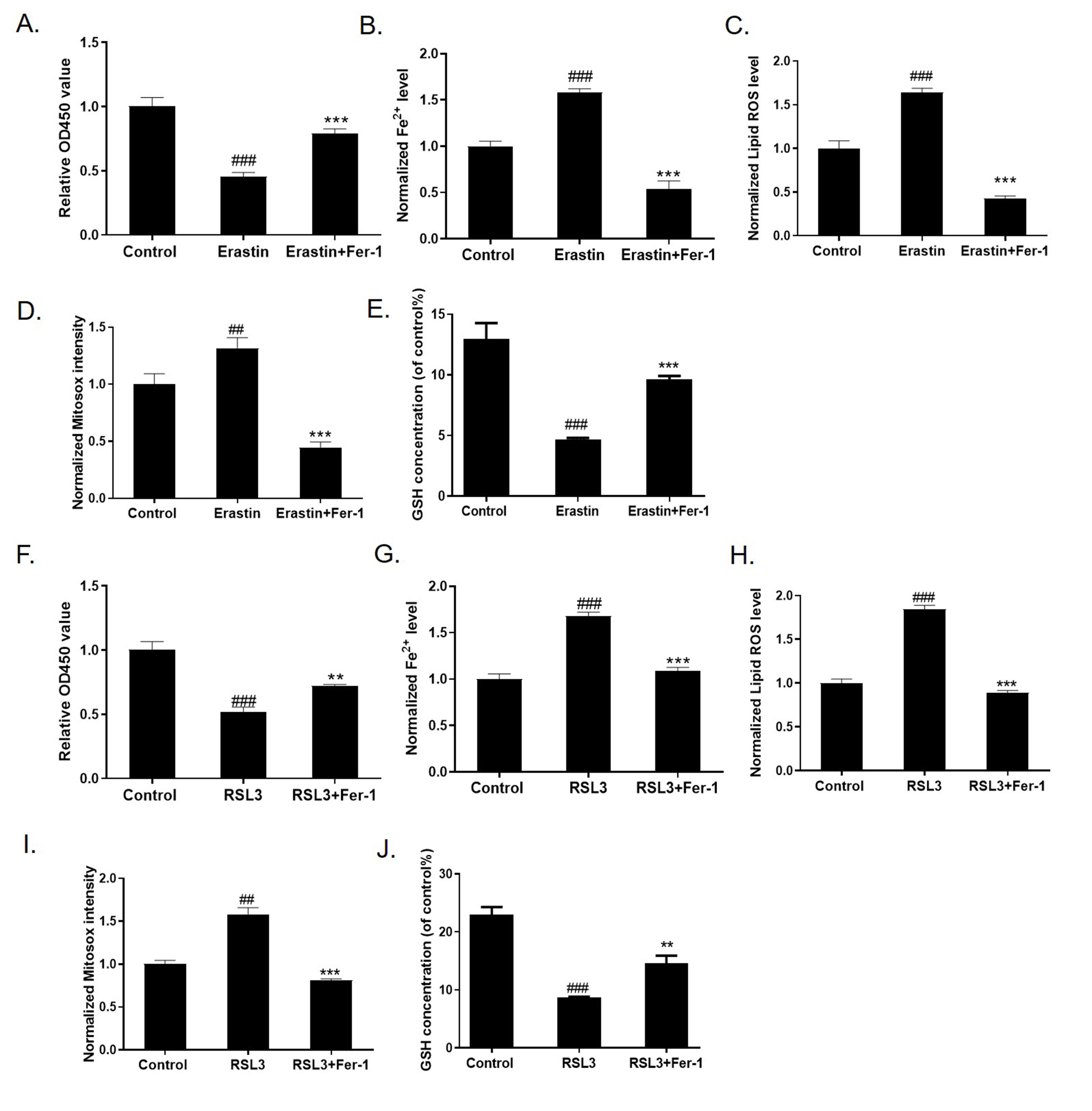

Supplement: Supplementary file 3 — Supplementary Figure1 [file 41419_2020_3135_MOESM3_ESM.tif]

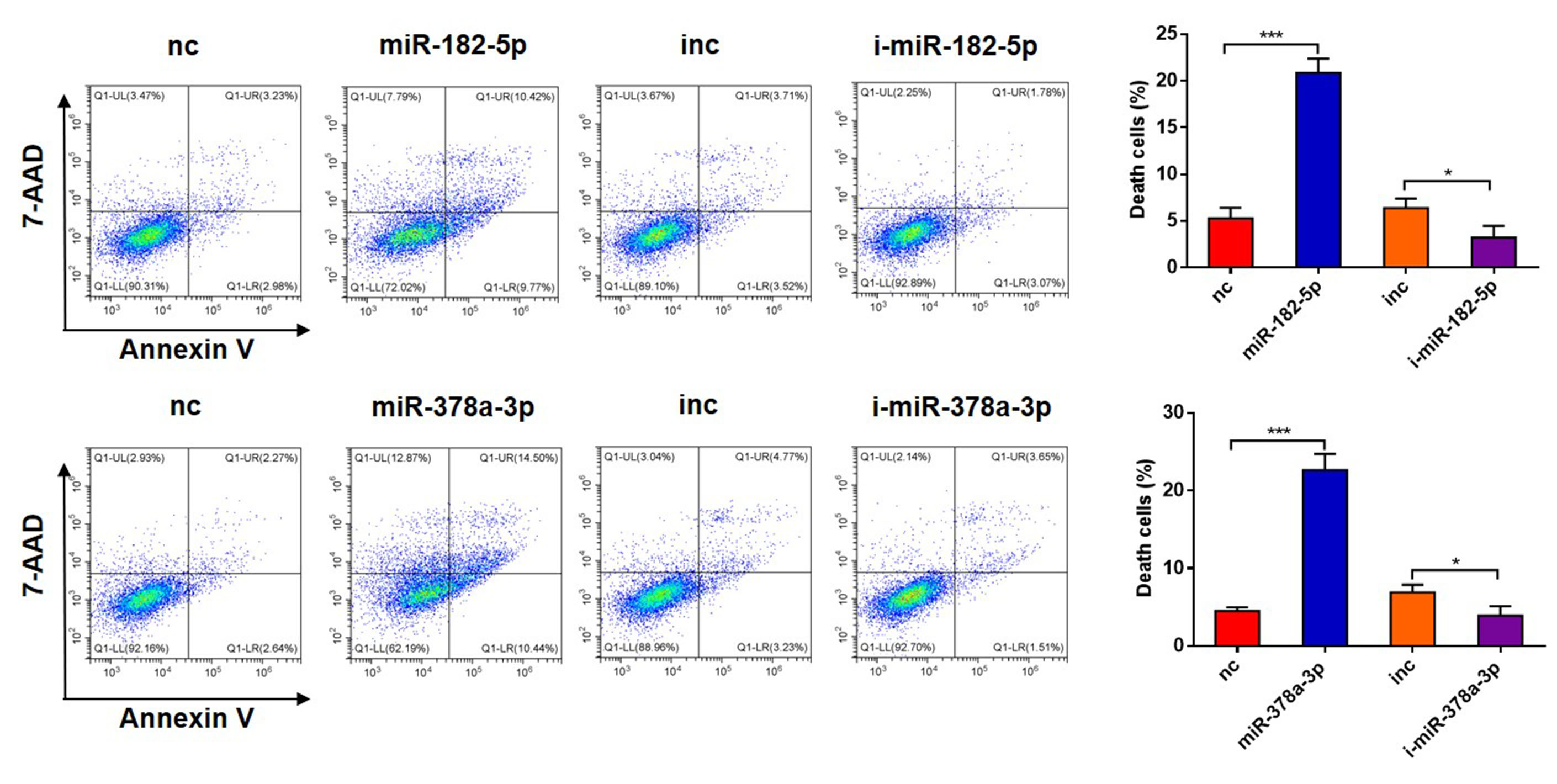

Supplement: Supplementary file 4 — Supplementary Figure2 [file 41419_2020_3135_MOESM4_ESM.tif]

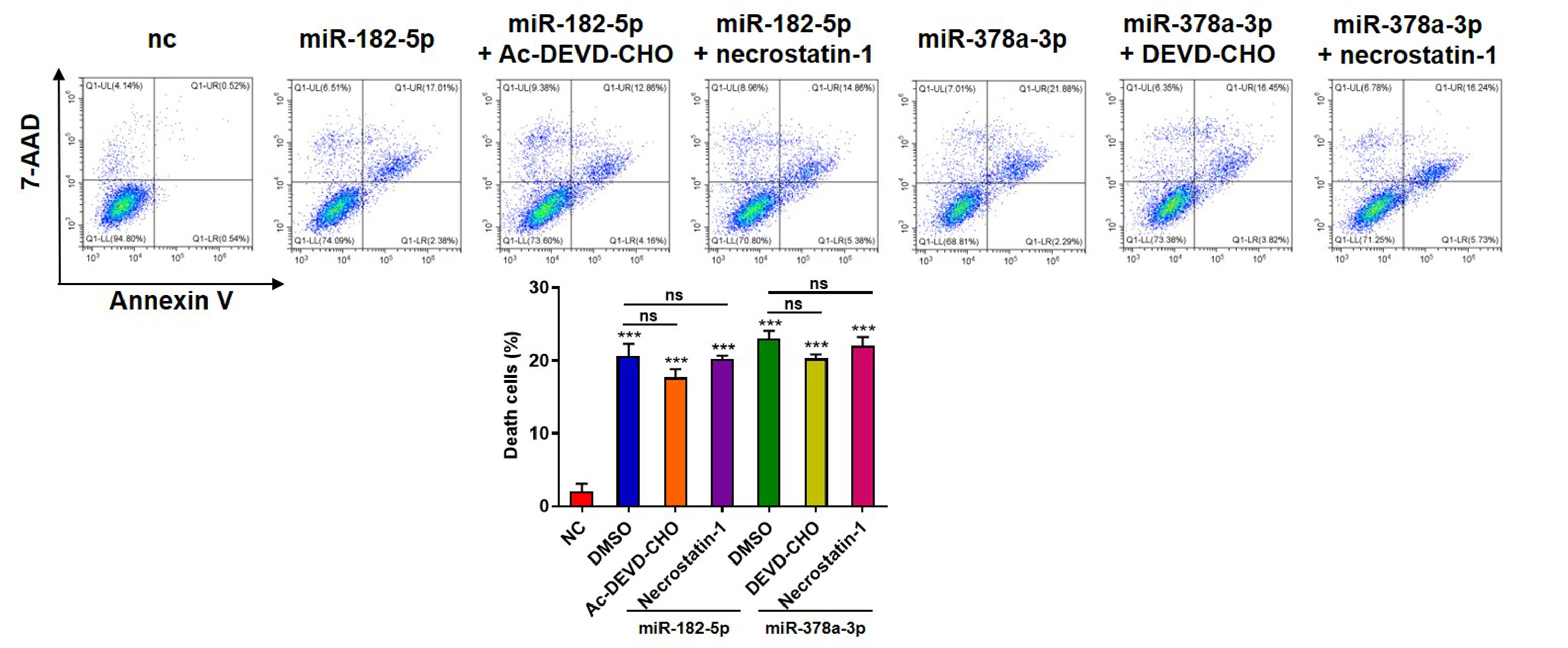

Supplement: Supplementary file 5 — Supplementary Figure3 [file 41419_2020_3135_MOESM5_ESM.tif]
